# Risk Assessment of Human Consumption of Meat From Fenbendazole-Treated Pheasants

**DOI:** 10.3389/fvets.2021.665357

**Published:** 2021-06-04

**Authors:** Marta Carreño Gútiez, Lisa A. Tell, Beatriz Martínez-López

**Affiliations:** ^1^Center for Animal Disease Modeling and Surveillance (CADMS), Department of Medicine & Epidemiology, School of Veterinary Medicine, University of California, Davis, Davis, CA, United States; ^2^Department of Medicine & Epidemiology, School of Veterinary Medicine, University of California, Davis, Davis, CA, United States

**Keywords:** food safety, risk analysis, drug residue, stochastic model, poultry, extra-label use

## Abstract

Fenbendazole is a benzimidazole-class anthelmintic that is used for the control of immature and adult stages of internal parasites, such as nematodes and trematodes, in domestic food-animal species. It is not approved by the United States Food and Drug Administration for treating pheasants despite *Syngamus trachea* being one of the most prevalent nematodes that parasitize pheasants. Because it is a highly effective treatment, e.g., 90% effectiveness against *S. trachea*, and there are very few alternative therapeutic options, this anthelminthic is used in an extra-label manner in the pheasant industry, but few studies have been conducted assessing risks to humans. Therefore, we conducted a risk assessment to evaluate the potential repeat-dose and reproductive, teratogenic, and carcinogenic human risks that may be associated with the consumption of tissues from pheasants that were previously treated with fenbendazole. We conducted a quantitative risk assessment applying both deterministic and stochastic approaches using different fenbendazole sulfone residue limits (tolerance, maximum residue limits, and analytical limit of detection) established in different poultry species by the Food and Drug Administration, the European Medicines Agency, and other regulatory agencies in Japan, Turkey, and New Zealand. Our results show that fenbendazole poses minimal risk to humans when administered to pheasants in an extra-label manner, and a comparison of different fenbendazole sulfone residue limits can help assess how conservative the withdrawal interval should be after extra-label drug use.

## Introduction

According to the United States (US) Food and Drug Administration (FDA), pheasants are considered to be “minor” food-animal species and categorized as game birds. Also, the United States Department of Agriculture census states that 7,790,734 pheasants were sold live in 2017 (1). Commercially raised game birds with parasitic infections are effectively treated with fenbendazole (2). Fenbendazole is a benzimidazole-class anthelmintic that is used for the control of immature and adult stages of some internal parasites, such as nematodes and trematodes in domestic food-animal species (3). Depending on the severity of the parasitic infection, morbidity and mortality can be quite high in pheasants, among other game birds (4). The most prevalent nematode in pheasants is *Syngamus trachea* (5). Two studies evaluating captive pheasants showed prevalences of 0.51 (6) and 0.37 (5) of *S. trachea*. The anthelmintic treatment currently approved for pheasants is thiabendazole (7), and both fenbendazole and thiabendazole have shown efficacy against adult and immature stages of some helminths. However, fenbendazole has a higher spectrum and lower dosage rates than thiabendazole in cattle (8). Also, early generation benzimidazoles, such as thiabendazole, have a lower margin of safety, are less specific and less potent than fenbendazole (9). Moreover, previous studies demonstrated that fenbendazole has up to 100% efficacy against a wide range of parasites (10) in cattle and more than 90% efficacy (2) against *S. trachea* in pheasants.

There are a few FDA-approved oral formulations of fenbendazole for food-producing animals in the United States of America (USA), such as Safe-Guard®Medication administration through feed, which is considered an effective and practical method of treatment for pheasants. Fenbendazole also seems to have higher efficacy when administered over several days (11). When consumed, fenbendazole is metabolized to fenbendazole sulfoxide and fenbendazole sulfone in turkeys (11), and the same metabolism is proposed to occur in pheasants.

The US FDA currently approves fenbendazole for use in turkey feed at a dose of 16 parts per million (ppm), but it is not yet approved for game birds in this country. In the United Kingdom, this drug is being used and approved in several game bird species at a dose of 12 ppm orally. In one study, fenbendazole was shown to be safe for Chinese Ring-Necked Pheasants when administered through feed at a dose of 100 ppm for 7 days (12). Due to a lack of FDA approval for administering fenbendazole to pheasants through medicated feed, it is prescribed in an extra-label manner which is allowed based on the regulatory discretion of an FDA inspector (13). However, withdrawal interval recommendations need to be established to ensure human food safety and long-term health. According to the FDA (14), the withdrawal period “is the interval between the time of the last administration of a new animal drug and the time when the animal can be safely slaughtered for food.” Thus, for an animal product to be marketable, the marker residue needs to be below a set target. Therefore, the FDA and European Medicines Agency (EMA) require drug sponsors to establish tolerance and maximum residue limit (MRL), respectively, as limits for every animal species and matrices (tissues, milk, eggs, or honey). When fenbendazole is administered to turkeys according to the FDA-approved label directions, the FDA-approved withdrawal period is zero days (15). Although tolerance has not been established for pheasants, in some cases, the FDA will allow the tolerance for a drug approved for use in a food-producing species to be extrapolated to the same drug for use in a food-producing minor species (16). Because the tolerance is dependent on the allowable daily intake (ADI) and marker residue, we chose the same marker residue for pheasants as chickens and turkeys based on the hypothesis that pheasants are similar in drug metabolism compared with other domestic poultry species (17, 18).

Therefore, according to the Food Animal Residue Avoidance Databank, the marker residue for pheasants has been assumed to be fenbendazole sulfone, and the target tissue is the liver, similar to turkeys (19), when estimating withdrawal intervals following extra-label drug use. In addition, since the drug is being used in an extra-label manner and no tolerance exists for pheasants, from a regulatory standpoint, the limit set as a maximum for the presence of residues is the assay's limit of detection (LOD) because it is a qualitative assessment for residue presence.

Having FDA-approved fenbendazole-medicated feed for pheasants could minimize the occurrence of resistance to antiparasitics, as it could facilitate the rotation of different antiparasitics. However, commercial pheasants are a relatively small commodity group compared with commercial chickens and turkeys. Therefore, drug sponsors have not pursued the additional label claim for pheasants. Given the common extra-label drug use of using fenbendazole to treat pheasants, there is a need to evaluate the human risks when consuming meat from pheasants treated with fenbendazole. This risk assessment can also help guide how conservative the estimated withdrawal recommendation should be to protect human health while still complying with established regulations to ensure no residue detection.

Therefore, the main objective of this study is to estimate the potential risks associated with repeat-dose toxicity, reproductive toxicity, teratogenicity, and carcinogenicity problems in humans that may arise from the prolonged consumption of pheasant meat from animals that were previously treated with fenbendazole. We conducted a quantitative risk assessment applying both deterministic and stochastic approaches and using different fenbendazole sulfone residue limits established in different poultry species by the FDA, the EMA, and other regulatory agencies in Japan, Turkey, and New Zealand.

## Materials and Methods

### Conceptual Model

**Figure 1:**
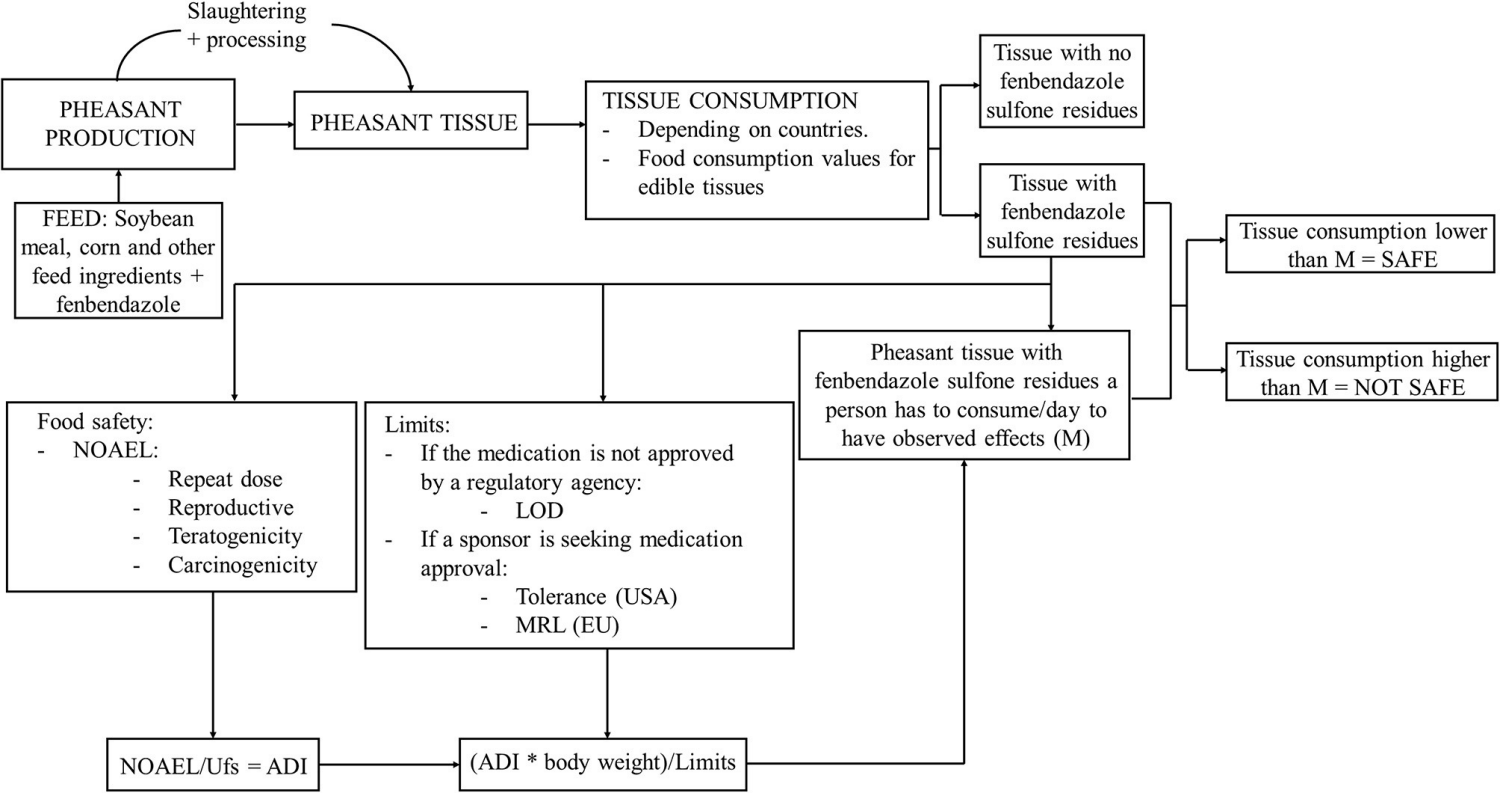
Conceptual framework summarizing risk assessment for humans consuming tissues from pheasants treated with fenbendazole-medicated feed. NOAEL, No-observed-adverse-effect levels; Ufs, standard uncertainty factor; ADI, acceptable daily intake; MRL, maximum residue limit; LOD, limit of detection.

Fenbendazole is an anthelmintic used to treat gastrointestinal nematodes in both small and large animals and is used to treat nematode parasitism in pheasants. Thus, when pheasants are slaughtered, fenbendazole sulfone residues may remain in the tissues. To analyze whether human consumption of pheasant tissues with fenbendazole sulfone residues is safe, we used both deterministic (i.e., using the most likely value for each parameter) and stochastic (i.e., including probability distributions) approaches considering diverse food safety adverse outcomes (repeat-dose toxicity, reproductive toxicity, teratogenicity, and carcinogenicity) and using different limits depending on approval status or if approval is being pursued (Figure 1).

We used the same risk assessment approach that is recommended by the World Organization for Animal Health (20) (OIE) and similar to other approaches used for food safety studies in the USA [e.g., United States Department of Agriculture Food Safety and Inspection Service Risk Assessment for Guiding Public Health-Based Poultry Slaughter Inspection (21)].

According to the EMA (22), fenbendazole produces low acute toxicity in mice, rats, rabbits, and dogs, and it is unknown if the final formulation (concentration of 20%) shows a skin-sensitizing property. In this study, fenbendazole did not show to have skin or eye irritating effects when administered at lower concentrations (concentration of 10%), and hypersensitivity reactions did not appear. However, this study was not made with the final formulation, so hypersensitivity reactions to fenbendazole or its excipients cannot be excluded. To evaluate systemic adverse effects, this study was performed on various animal species. Their findings in toxicological studies in mice, rats, rabbits, and dogs demonstrated some no-observed-adverse-effect levels (NOAELs) for different toxicities. The study made in rats and dogs discovered that the NOAEL for repeat-dose toxicity was 4 mg/kg, and dogs showed lymphoid hyperplasia. Also, a NOAEL of 15 mg/kg was established in a reproductive toxicity study in rats, as they had symptoms such as reductions in fertility, survival, and growth of the neonates during lactation. Moreover, a NOAEL of 25 mg/kg was established for rabbits in the teratogenicity study based on an increase in delayed ossification. Because fenbendazole has no structural alerts for carcinogenicity and evidence for carcinogenicity has not been found, there are no data on carcinogenicity. However, a carcinogenicity study performed in rats by the World Health Organization (WHO) (23) established a NOAEL of 0.7 mg/kg showing hepatocellular lipid vacuolation.

As fenbendazole is a veterinary medication, as indicated by the EMA (22), this product is limited to being prescribed by veterinarians. Besides the consumption of residue-contaminated animal products, farmers distributing medicated feed to animals can get exposed through ocular and dermal contact. However, here, we will focus mostly on the potential risk of human exposure through the consumption of pheasant meat with fenbendazole sulfone residues.

### Input Data

Because no data for pheasant meat consumption in the USA are available, the population used for this study were production turkeys consumed in the USA obtained from the National Chicken Council (24) because the metabolism of fenbendazole in turkeys is suspected to be very similar to the metabolism in pheasants, and turkeys are a commodity group consumed at lower rates compared with chickens. Also, as there is a lack of pheasant meat consumption data and fenbendazole pheasant tissue residue limit data, we have assumed data from other species to estimate the human risk of pheasant meat consumption with fenbendazole sulfone residues. In addition, data regarding pheasant fresh meat consumed in the European Union (EU) were obtained from the WHO (25). These data showed that the country with the highest consumption of pheasant fresh meat in the EU was Belgium. In addition, there was information for pheasant fresh meat consumed by the total population in Belgium and pheasant fresh meat consumption among consumers in Belgium, so both data were taken for our study to present the “worst-case” scenario. Turkey consumption data in the USA and pheasant fresh meat consumption data in Belgium were taken from 2019 and 2004 censuses, respectively, as those were the most recent years for which data was complete. Furthermore, the 2018 FDA guidance for calculating the withdrawal period (14) and EMA guidelines (26) reported some food consumption values for edible tissues. When FDA estimates the daily consumption for edible tissues, it assumes that a person would not consume any portion from any other animal species on the same day. Values for turkey consumption in the USA, pheasant consumption in the entire EU population and among pheasant consumers in Belgium, and food consumption values for edible tissues according to the FDA guideline are provided in Table 1.

**Table 1:** Turkey meat consumption data in USA (2019), pheasant fresh meat consumption data in Belgium (2004), and FDA/EMA food consumption values for edible tissues (2018).

**Species**	**Country**	**Tissue**	**Mean consumption values (g/day)**	**Reference**
Turkey	USA	Muscle	119.55	National Chicken Council (24)
Pheasants	Belgium	Muscle	0.33 (mean = 76.37 and 95 percentile = 119.01 for pheasant consumers)	WHO (25)
All food	USA-EU	Muscle	300	FDA (14)
	USA-EU	Liver	100	FDA (14)

Data regarding fenbendazole sulfone residue concentration limits were extracted from both FDA (19) and EMA (22) reports. To calculate the worst-case scenario, tolerances (FDA) and MRLs (EMA) were used, as animals should have tissue residue concentrations lower than the tolerance or MRL to be slaughtered or consumed. Therefore, FDA- or EMA-approved withdrawal periods are established using tolerance or MRL to ensure that the human consumption of tissue is safe if it contains fenbendazole sulfone residues. There are different established FDA-approved tolerances for several species and matrices. The FDA and the EMA make distinctions in tolerances for different matrices (liver, meat, milk, and eggs) due to differences in consumption by humans. However, the EMA establishes the same MRL for oxfendazole sulfone for all food-producing species except fish, as *in vivo*, fenbendazole mainly exists in its oxidized oxfendazole form. The tolerances and MRLs used for our study were liver residue limits from different avian species, as the liver is the target tissue for fenbendazole sulfone in turkeys (19). Furthermore, previously published analytical LODs for pheasant edible tissues were used in this study to calculate a more realistic scenario (12), as US regulatory guidelines mandate that residues should not be detectable if there is no tolerance. Also, duck liver tolerances from countries that are not part of the USA or the EU (27) were included to analyze a wider range of tolerances. The different liver tolerances and MRLs and pheasant tissue LODs used in this study are summarized in Table 2.

**Table 2:** FDA liver tolerances, EMA liver MRLs, pheasant's fenbendazole sulfone LODs and Japan, New Zealand, and Turkey duck liver tolerance for different tissues.

**Species**	**Country/region**	**Tissue**	**Criteria/reference limits**	**Value (ppm)**	**Reference**
Turkey	USA	liver	Tolerance	6	FDA (19)
Chicken	USA	liver	Tolerance	5.2	FDA (19)
All species	Europe	liver	MRLs	0.5	EMA (22)
Pheasant	USA	liver	LOD	0.04	Pheasant safety study (12)
Pheasant	USA	pectoral	LOD	0.004	Pheasant safety study (12)
Pheasant	USA	thigh	LOD	0.005	Pheasant safety study (12)
Duck	Japan	liver	Tolerance	2	Kansas State University (KSU) (27)
Duck	New Zealand	liver	Tolerance	0.1	KSU (27)
Duck	Turkey	liver	Tolerance	0.05	KSU (27)

*MRL, maximum residue limit; LOD, Limit of detection*.

The human food consumption risk assessment was developed and implemented using @Risk version 7.5.1 (Palisade®, Ithaca, US) in Microsoft Excel. Some results for the stochastic risk assessment were displayed using box plots generated using R-language (28) and RStudio version 1.2.5001, implementing “dplyr” and “ggplot2” packages.

### Risk Assessment Model

#### Deterministic Approach: Calculations per Country and Tissue

We first used a deterministic approach to quantify the degree of human risk of repeat-dose toxicity, reproductive toxicity, carcinogenicity, and teratogenicity observed adverse effects by consuming tissue from pheasants previously treated with fenbendazole using the current regulatory values per country and tissue.

Several steps were used to calculate how many grams of pheasant tissue with fenbendazole sulfone residues/day a person has to consume to have different observed adverse effects. First, according to the FDA (29), the acceptable daily intake was calculated by multiplying the NOAELs of the different possible adverse effects by a standard uncertainty factor (Ufs) Equation (1). The NOAELs used for these calculations were taken from the EMA (22). NOAELs for repeat-dose toxicity, reproductive toxicity, and teratogenicity effects were 4, 15, and 25 mg/kg bw/day, respectively. Also, the EMA did not observe any carcinogenetic effects regardless of the dose of fenbendazole used. However, a rat-model study from the WHO (23) was used to evaluate the worst-case scenario for carcinogenetic effects using a NOAEL of 0.7 mg/kg bw/day.

(1) $$\begin{array}{l} ADI\;\left(mg/kg\;bw/day\right)\;=\;NOAEL\;\left(mg/kg\;bw/day\right)\times Ufs \end{array}$$

The repeat-dose, reproductive, carcinogenicity, and teratogenicity NOAELs were used for the deterministic process. Also, three standard uncertainty factors were evaluated: 10, 100, and 1,000. FDA states that a standard uncertainty factor of 100 is recommended. However, Ufs of 10 and 1,000 were also included in the deterministic process to evaluate worst- and best-case scenarios.

Taking the FDA assumption of 60 kg of body weight per person (30), the ADI for a 60 kg person was calculated Equation (2). Because we wanted to create the worst-case scenario, we used the FDA assumption of 60 kg, as it is a lower body weight compared with the European and American body weight means.

(2) $$\begin{array}{lll} ADI\;for\;60\;kg\;\left(mg/day\right)\; & = & \;ADI\;\left(mg/kg\;bw/day\right)\\ \times \;60\;kg\;bw \end{array}$$

Finally, we estimated the daily amount of pheasant tissue a person has to consume to have different observed adverse effects due to the presence of fenbendazole sulfone residues in pheasant tissue Equation (3):

(3) $$\begin{array}{l} Amount\;of\;pheasant\;tissue\;with\;fenbendazole\;sulfone\;residues\\ a\;person\;has\;to\;consume/day\;to\;have\;observed\;adverse\\ effects:\;=\;\frac{ADI\;for\;60\;kg\;\left(mg/day\right)}{Tolerance\;or\;MRL\;or\;LOD} \end{array}$$

The nine different limits that were used for the deterministic process are shown in Table 2.

Estimates of grams of pheasant tissue with fenbendazole sulfone residues that would need to be consumed daily to observe adverse effects of repeat-dose toxicity, reproductive, carcinogenicity, and teratogenicity problems, depending on the Ufs and on limits established by different countries in different poultry species and tissues, were determined.

After estimating the amount of pheasant tissue with fenbendazole sulfone residues that have to be consumed to have observed adverse effects Equation (3), we evaluated if human consumption of tissue with fenbendazole sulfone residues was safe. To know if the consumption of pheasant tissue from animals that were previously treated with fenbendazole was safe for humans, these calculations were compared with the actual consumption data in the USA and Belgium (EU) and to food consumption values for edible tissues established by the FDA/EMA and using all the fenbendazole sulfone NOAELs (repeat-dose, reproductive, carcinogenicity, and teratogenicity) (Table 1) and all the established limits (Table 2). Finally, to decide if tissue consumption was safe, the previously calculated amount of pheasant tissue with fenbendazole sulfone residues a person has to consume daily to have observed adverse effects by country and Ufs was divided by different fenbendazole sulfone residue limits. Results that were ≥1 were assumed not to be safe, as there could be adverse effects related to the consumption of tissue from pheasants previously treated with fenbendazole. Results that were <1 were assumed to be safe.

#### Stochastic Approach: Overall Risk Independently of Country or Tissue

In addition to the deterministic approach, we combined geographic regional consumptions and tissues into one single stochastic model to estimate the overall risk associated with consumption of pheasant tissue with fenbendazole sulfone residues independent of the country of origin of the tissue or type of tissue consumed. For this stochastic model, probability distributions were used to capture the variability and uncertainty associated with tissue consumption for different regions and multiple tissues (Table 3). In addition, three different scenarios were developed to consider the three different criteria or reference limits: tolerance, MRL, and LOD. The different established tolerances for liver from chicken and turkey in the USA and for liver from duck in Turkey, Japan, and New Zealand were integrated into a uniform distribution, using the duck liver tolerance (Turkey) as a minimum (50 parts-per-billion [ppb] or 0.05 ppm) and the USA turkey liver tolerance as a maximum (6,000 ppb or 6 ppm) (Table 3). The established MRL by the EMA values were used to create a Pert distribution (Table 3). A second Pert distribution was created using different pheasant tissue LODs: the LOD of 0.004 ppm (pectoral muscle) as a minimum, the LOD of 0.005 ppm (thigh muscle) as the most likely value, and the LOD of 0.04 ppm (liver) as a maximum (19).

**Table 3:** Summary of parameters used for stochastic process to perform a risk assessment for human consumption of pheasant tissues with fenbendazole sulfone residues.

**Input variable**	**Distribution**	**Values**	**Source**
NOAELs	Repeat-dose = Normal (mean, sd)	Mean = 4 mg/kg bw/daySd = 0.4	EMA (22)
	Reproductive = Normal (mean, sd)	Mean = 15 mg/kg bw/daySd = 1.5	
	Teratogenicity = Normal (mean, sd)	Mean = 25 mg/kg bw/daySd = 2.5	
	Carcinogenicity = Normal (mean, sd)	Mean = 0.7 mg/kg bw/daySd = 0.07	WHO (23)
Kg bodyweight	Pert (min, most likely, max)	Minimum = 50 kg,most likely = 70.8, maximum = 129 kg	NHANES (31), Weight of nations (32)
Ufs	Normal (mean, sd)	Mean = 100Sd = 10	FDA (14)
Limits	Tolerance = Uniform (minimum, maximum)	Minimum = 0.00005 mg/g [Duck liver tolerance (Country: Turkey)]	KSU (27)
		Maximum = 0.006 mg/g [Turkey liver tolerance (FDA)]	FDA (19)
	MRL = Pert (minimum, most likely, maximum)	Minimum = 0.00045 mg/gMost likely = 0.0005 mg/gMaximum = 0.00055 mg/g	EMA (22)
	LOD = Pert (minimum, most likely, maximum)	Minimum = 0.000004 mg/g (pectoral)Most likely = 0.000005 mg/g (thigh)Maximum = 0.00004 mg/g (liver)	Pheasant safety study (12)
Pheasant consumption	USA = Pert (minimum, most likely, maximum)	Minimum = 100 g/day (liver)Most likely = 119.55 g/day (turkey tissue consumption)Maximum = 300 g/day (muscle)	FDA (14) National Chicken council (24)FDA (14)
	EU = Pert (minimum, most likely, maximum)	Minimum = 0.33 g/day (pheasant fresh meat, all consumers in Belgium)Most likely = 76.37 g/day (pheasant fresh meat consumption mean, consumers in Belgium)Maximum = 119.01 g/day (pheasant fresh meat, percentile 95, consumers in Belgium)	WHO (25)

*NOAEL, No-observed-adverse-effect levels; Ufs, Standard Uncertainty Factor; MRL, maximum residue limit; LOD, limit of detection*.

According to the FDA, the Center for Veterinary Medicine, the reference center for animal food and feeds regulation in the USA, has historically applied a safety factor of 100 for an ADI based on the NOAEL (14); hence, a normal distribution for a standard Ufs of 100 was used for this stochastic process. Also, NOAELs for repeat-dose, reproductive, carcinogenicity, and teratogenicity problems were used to create normal distributions. The human weight (i.e., kg of body weight) was modeled using a Pert distribution with a minimum of 50 kg [percentile 5 for men in the USA (31)], a most likely value of 70.8 kg, based on the European average (32) and a maximum of 129 kg [percentile 95 for men in the USA (31)]. All distributions were truncated using ±3 standard deviation values, and the number of iterations was 5,000. The same model applied for the deterministic process Equations (1–3) was used for the stochastic process but applying the different distributions for each parameter (Table 3). The calculations made with the distributions of tolerances and MRLs were made to evaluate the worst-case scenarios, and all results are expressed as 95% confidence intervals.

Thus, applying the mathematical model previously created Equation (3), a range of grams a day of pheasant tissue that has to be consumed to have observed adverse effects was calculated using tolerance, MRL, and LOD distributions.

A sensitivity analysis for the LOD distribution and repeat-dose NOAEL was performed to evaluate the impact that changes in input values have on the number of pheasant tissues with fenbendazole sulfone residues a person has to consume a day to have observed adverse effects. Thus, a tornado graph was created, and correlation coefficients applying Spearman rank were calculated to carry out the sensitivity analysis. LOD distribution and repeat-dose NOAEL were chosen, as they were the most conservative parameters not considering the carcinogenicity NOAEL because no evidence for carcinogenicity was found by the EMA (22).

Finally, to evaluate if human consumption of pheasant tissues with fenbendazole sulfone residues is safe, we used the same procedure that was described for the deterministic approach: US and EU consumptions based on the distributions were divided into the range of pheasant tissues a person has to consume daily to have observed adverse effects. Thus, results with a mean <1 were considered safe, and results with a mean ≥1 were considered unsafe.

In addition, turkey tissue consumption per capita in the USA was combined with the established consumption for edible tissues according to the FDA and EMA and modeled using a Pert distribution: the most likely value was 119.55 g/day [turkey consumption per capita in the US (24)], 100 g/day was used as the minimum (liver consumption by the FDA/EMA), and 300 g/day as the maximum (muscle consumption by the FDA/EMA) (14). Furthermore, pheasant fresh meat consumption in the EU was modeled using a Pert distribution using the average daily consumption of pheasant fresh meat in Belgium (0.33 g/day) as the minimum, the most likely value using the average daily consumption of pheasant fresh meat among consumers of this meat in Belgium (76.37 g/day) and as the maximum the 95th percentile of consumption of pheasant fresh meat among consumers of this meat in Belgium (119.01 g/day) (25).

## Results

### Pheasant Tissue Consumption With Fenbendazole Sulfone Residues to Have Observed Adverse Effects and Evaluation of the Safety of This Human Food Consumption

#### Deterministic Model Results

We calculated the different amounts of pheasant tissue with fenbendazole sulfone residues that had to be consumed to observe adverse effects based on different limits according to the country, species, and tissues (Table 4). It can be observed that the limit that allows a lower intake of pheasant consumption is the turkey liver tolerance in the USA, as its tolerance is the highest one. On the other hand, LODs allow a greater tissue intake, as their limits are very low and, therefore, safer. As expected, the higher the Ufs is applied, the lower the amount of pheasant tissue with fenbendazole sulfone residues can be daily ingested to show different adverse effects.

**Table 4:** Grams/day of previously treated with fenbendazole poultry tissue a person has to consume to have observed adverse effects.

**Tissue consumption to have observed effects (g/day)**						
		**Effect**	**Repeat dose**	**Reproductive**	**Teratogenicity**	**Carcinogenicity**
**Tissue-Ufs**	**Species**	**Country-reference limit**				
Liver	Chicken	USA-Tolerance				
Ufs = 10			4,615	17,308	28,846	808
Ufs = 100			462	1,731	2,885	81
Ufs = 1,000			46	173	288	8
Liver	Turkey	USA-Tolerance				
Ufs = 10			4,000	15,000	25,000	700
Ufs = 100			400	1,500	2,500	70
Ufs = 1,000			40	150	250	7
Liver	Duck	Japan-Tolerance				
Ufs = 10			12,000	45,000	75,000	2,100
Ufs = 100			1,200	4,500	7,500	210
Ufs = 1,000			120	450	750	21
Liver	Duck	New Zealand-Tolerance				
Ufs = 10			240,000	900,000	1,500,000	42,000
Ufs = 100			24,000	90,000	150,000	4,200
Ufs = 1,000			2,400	9,000	15,000	420
Liver	Duck	Turkey-Tolerance				
Ufs = 10			480,000	1,800,000	3,000,000	84,000
Ufs = 100			48,000	180,000	300,000	8,400
Ufs = 1,000			4,800	18,000	30,000	840
Liver	All	EU-MRL				
Ufs = 10			48,000	180,000	300,000	8,400
Ufs = 100			4,800	18,000	30,000	840
Ufs = 1,000			480	1,800	3,000	84
Liver	Pheasant	USA-LOD				
Ufs = 10			600,000	2,250,000	3,750,000	105,000
Ufs = 100			60,000	225,000	375,000	10,500
Ufs = 1,000			6,000	22,500	37,500	1,050
Pectoral muscle	Pheasant	USA-LOD				
Ufs = 10			6,000,000	22,500,000	37,500,000	1,050,000
Ufs = 100			600,000	2,250,000	3,750,000	105,000
Ufs = 1,000			60,000	225,000	375,000	10,500
Thigh muscle	Pheasant	USA-LOD				
Ufs = 10			4,800,000	18,000,000	30,000,000	840,000
Ufs = 100			480,000	1,800,000	3,000,000	84,000
Ufs = 1,000			48,000	180,000	300,000	8,400

*MRL, maximum residue limit; LOD, limit of detection*.

When evaluating all the results obtained after performing the deterministic analysis of the human safety of consuming pheasant tissues with fenbendazole sulfone residues by applying three different Ufs, we found that in very few cases, those values were considered unsafe (i.e., values > 1) (Supplementary Tables 1, 2). Furthermore, when applying a Ufs of 10, all pheasant tissue consumption seems to be safe, and no adverse effects are going to be observed. Furthermore, considering the values corresponding to a Ufs of 100, it can be observed that the vast majority can be considered safe, although there are some values corresponding to the NOAEL of carcinogenicity that is not safe. Moreover, the limit with the highest number of values >1 corresponds to the turkey liver tolerance in the USA. This is due to the fact this tolerance is higher in this study (6 ppm). Also, when applying a more realistic scenario, that is, analyzing the safety with the LODs, it can be observed that all values are lower than one even with a Ufs of 1,000; hence, daily pheasant tissue consumption with residues equal to the LOD, independently of the tissue, seems to be safe.

#### Stochastic Model Results

Our stochastic risk assessment model revealed that the mean amount of tissue consumption per day that a person has to have to observe adverse effects using the pheasant LOD distribution and the repeat-dose NOAEL is 382,965.14 g/day ([122,955.85, 859,487.58] g/day, 95% confidence interval) (Supplementary Figure 1).

Moreover, the highest value of pheasant tissue consumption a person has to eat daily to have observed adverse effects, 2,281,479.10 g/day, corresponds to the pheasant LOD distribution [which has the lowest most likely value for the limit distributions that were analyzed (0.000005 mg/g)] and teratogenicity NOAEL, which is the highest NOAEL (25 ppm) (Supplementary Table 3).

**Figure 2:**
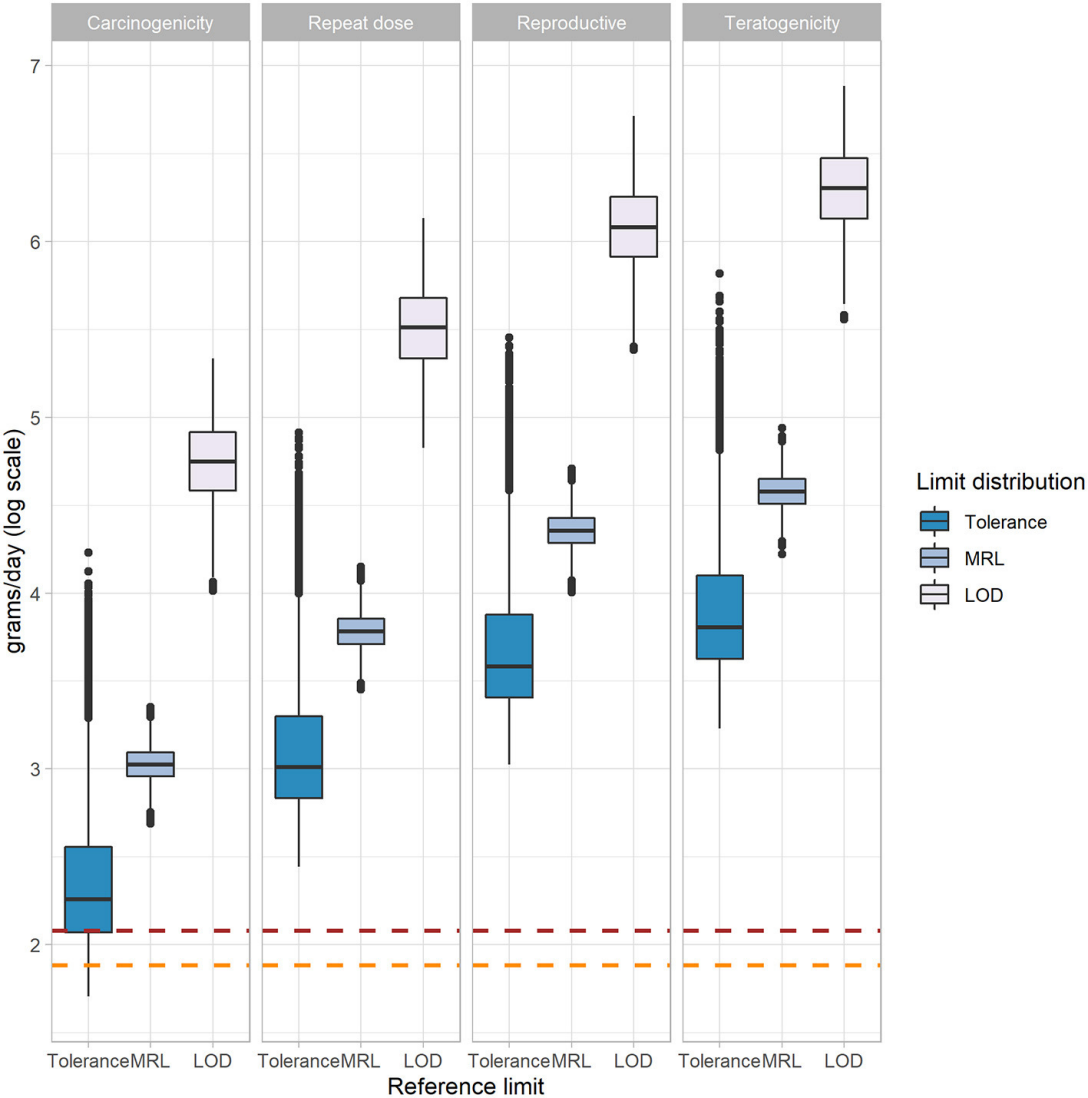
Comparison of three stochastic processes applying tolerance, MRL, and LOD distributions for four different NOAELs in a box plot to calculate human consumption (log grams/day) of pheasant tissues contaminated with fenbendazole sulfone residues to have observed adverse effects. Boxplots are visualized using minimum (Q1 − 1.5 × IQR), first quantile (Q1), median (Q2), third quartile (Q3), and maximum values (Q3 + 1.5 × IQR). IQR is interquartile range. Outliers are represented using points. Brown dotted line, log most likely value for US consumption distribution (119.55 g/day); orange dotted line, log most likely value for EU consumption distribution (76.37 g/day); MRL, maximum residue limit; LOD, limit of detection; NOAEL, No-observed-adverse-effect level.

Also, as the reference limit has a smaller value, tissue consumption may be higher (Figure 2). Therefore, when carrying out the calculations with the pheasant LOD distribution, which has the smallest most likely value (0.000005 mg/g), tissue consumption will be higher to observe adverse effects due to consumption of pheasant tissue with fenbendazole sulfone residues. The opposite occurs with the tolerance distribution, as it has the highest cutoff; therefore, pheasant tissue consumption will be lower until adverse effects are observed.

**Figure 3:**
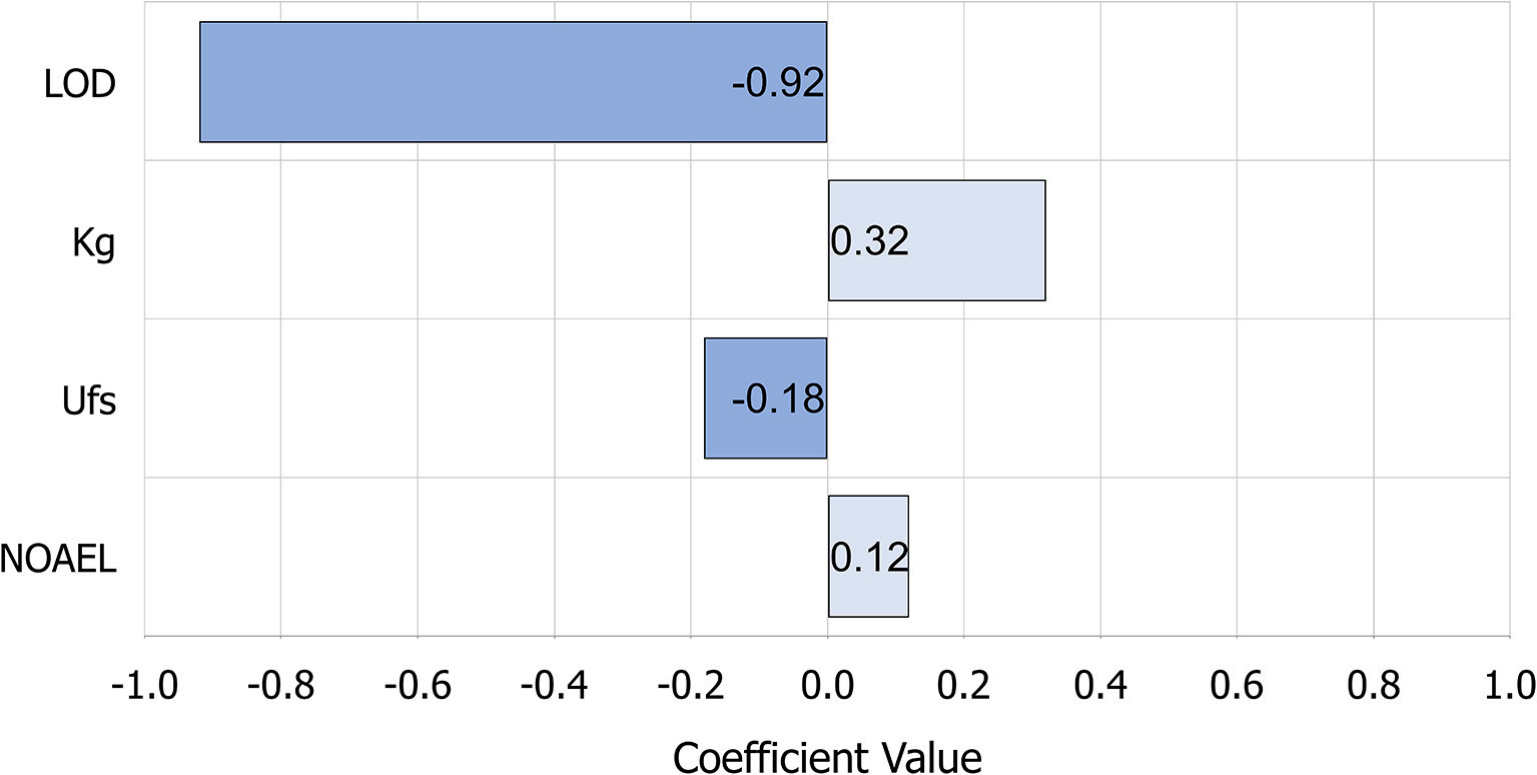
Sensitivity analysis applying Spearman's rank correlation coefficient of having adverse effects due to repeated dose of consumption of pheasant tissues with fenbendazole sulfone residues using pheasant LOD distribution. LOD is very strong negatively correlated; kilograms of body weight is moderate positively correlated; Ufs relationship is negligible; NOAEL relationship is negligible. Ufs, standard uncertainty factor; LOD, limit of detection; NOAEL, No-observed-adverse-effect level.

A sensitivity analysis was also performed with the pheasant LOD distribution to know which factor has the highest influence on having adverse effects due to repeated dose (Figure 3). As a result of this sensitivity analysis, we found that the factor with the strongest influence on having observed effects after consuming pheasant tissues in this case is the LOD, which is very strong negatively correlated (−0.91). The rest of the elements of the model have a much lower correlation, being the NOAEL and the kilograms of body weight positively correlated, and the Ufs negatively correlated with the amount of pheasant tissue a person has to eat to have observed adverse effects.

Overall, our stochastic model found that the consumption of pheasant tissues with fenbendazole sulfone residues is safe (Table 5). Because all values in Table 5 are multiplied by 10^2^, results ≥100 express that the consumption of pheasant tissues in the USA and the EU is not safe, and results lower than 100 express that it is safe even when having fenbendazole sulfone residues according to the distributions of the tolerances, MRLs, and LODs.

**Table 5:** Human food safety of consuming poultry tissues with fenbendazole sulfone residues applying a Ufs of 100.

**Human food safety of consuming pheasant tissues applying a Ufs=100 (mean [95% confidence interval])**				
	**Effect**	**Tolerances**	**MRLs**	**LODs**
USA consumption	Repeat dose	14.86 [0.90, 36.95]	2.45 [1.41, 4.05]	0.05 [0.02, 0.13]
	Reproductive	3.96 [0.24, 9.85]	0.65 [0.38, 1.08]	0.01 [<0.01, 0.03]
	Teratogenicity	2.38 [0.14, 5.91]	0.39 [0.23, 0.65]	0.01 [<0.01, 0.02]
	Carcinogenicity	84.90 [5.14, ***211.13***]	14.02 [8.08, 23.15]	0.30 [0.09, 0.75]
EU consumption	Repeat dose	7.18 [0.39, 18.86]	1.19 [0.40, 2.11]	0.03 [0.01, 0.07]
	Reproductive	1.91 [0.10, 5.03]	0.32 [0.11, 0.56]	0.01 [<0.01, 0.02]
	Teratogenicity	1.15 [0.06, 3.02]	0.19 [0.06, 0.34]	<0.01 [<0.01, 0.01]
	Carcinogenicity	41.02 [2.24, ***107.76***]	6.79 [2.29, 12.03]	0.14 [0.03, 0.38]

*All numbers are multiplied by 10^2^. Values in bold mean that consumption is unsafe (≥100)*.

Again, the obtained results after performing the stochastic analysis to determine the human safety of the consumption of pheasant tissue with fenbendazole sulfone residues show that human consumption is safe, applying almost all consumption distributions and limits studied for the different adverse effects that may arise due to the consumption of the different tissues, as the means are lower than 100. However, in the case of possible adverse effects related to carcinogenicity that may arise as a result of the human consumption of these tissues, it is observed that by applying the uniform distribution of the tolerances, this consumption may not be safe. On the one hand, the mean for the safety value of presenting carcinogenicity adverse effects is lower than 100 when applying the distributions of the US and EU consumptions in all the limit distributions. However, applying both consumption distributions (the USA and European consumption), even if the 97.5 percentile is higher than 100, and using the tolerances distribution as a limit, it cannot be concluded that consumption of pheasant tissues with fenbendazole sulfone residues is unsafe, as both confidence intervals include a value lower than 100. Also, it is observed that the lowest numbers, and therefore the safest cases, correspond when applying the distribution of the LODs and the NOAEL of teratogenicity. This seems reasonable, as the limits for the LODs are the lowest, and teratogenicity adverse effects have the highest NOAELs.

## Discussion

This study aimed to estimate the risk associated with human consumption of tissues obtained from fenbendazole-treated pheasants. To the best of the authors' knowledge, this is the first study conducted for this drug and this specific species. We evaluated multiple scenarios using both deterministic and stochastic approaches. On the one hand, the deterministic model offers specific results for different countries that establish their own limits for human consumption of pheasant tissues with residues of this drug. Thus, this adds the advantage that, in addition to the ranges already provided with the stochastic process, results from different countries can also be observed, which could also help for safe extra-label drug use of fenbendazole in pheasants in more countries apart from the USA and the EU. Also, our model contributes to consider different Ufs in addition to the 100 Ufs established by the FDA (14), which is interesting to carry out other types of assessments that require Ufs of 10 or 1,000. In addition, applying the different NOAELs of repeat-dose toxicity, reproductive toxicity, teratogenicity, and carcinogenicity from previous studies carried out in different species (22), our study contributes to determining if, after human consumption of pheasant tissue with fenbendazole sulfone residues, there are problems in humans that may arise from the prolonged consumption of pheasant tissue from animals that were previously treated with fenbendazole. Moreover, our approach of visualizing whether the score obtained by dividing the actual pheasant and other poultry tissue consumption by the amount that must be consumed to present different adverse effects is greater or equal, or less than one, it is a simple and quick way of being able to know if human consumption of pheasant tissues with fenbendazole sulfone residues is safe. On the other hand, the stochastic model is very useful for evaluating the overall safety of consumption of pheasant tissue with fenbendazole sulfone residues independently of region and tissue consumed and taking into account the variability and uncertainty associated with various factors (e.g., body weight, amount of tissue consumption, limit values used, etc.). Therefore, it is very useful to obtain a range of values that take into account all possible scenarios to be able to evaluate whether or not the consumption of pheasant tissue with fenbendazole sulfone residues is safe. Another method by which the safety of consuming pheasant tissue with fenbendazole sulfone residues can be ascertained is by comparing the human consumption of tissue that should be consumed daily to observe effects with the actual consumption of pheasant tissue in each country. However, this is difficult to do due to the scarcity of statistics about pheasant tissue consumption, and this method is more cumbersome, as the data would have to be compared one by one to know the safety of the human consumption of pheasant tissue.

When looking at tissue consumption values applying a Ufs of 10 in Table 4, it can be observed that the minimum consumption corresponds to applying the turkey liver tolerance in the USA, as this limit is the highest cutoff in our study (6 ppm), and the NOAEL of carcinogenicity, which corresponds to daily consumption of 700 g of pheasant tissue to have these adverse effects. However, the EMA assumes that carcinogenicity effects have not been observed, and the WHO states that they are very unlikely. Nevertheless, taking into account these data, the consumption of pheasant tissue that should be consumed to have carcinogenicity effects is very high and exceeds by far the average consumption of pheasant tissue of the population. Using the stochastic process, the means of pheasant tissue with fenbendazole sulfone residues consumption a person has to eat to have observed adverse effects with the most conservative NOAEL, that is, the carcinogenicity NOAEL (0.7 mg/kg bw/day) was 441.91 g a day applying the tolerances distribution limit, the highest one. Moreover, 2,281,479.10 g a day of pheasant tissue should be consumed to have observed effects when applying the pheasant LOD distribution and teratogenicity NOAEL, which are both the less conservative limits and NOAEL distributions in this study (Supplementary Table 3). This is an extremely high level of consumption, and it is unlikely to occur. Also, comparing the calculations made with the MRLs and the tolerances (Supplementary Table 3), the EMA-based tissue consumption with fenbendazole sulfone residues to have adverse effects calculation is higher than the one applying the tolerances because the EMA MRLs are more conservative than the tolerances used in this study. Therefore, after performing all the stochastic analyses and according to data, it can be deduced that the amount of tissue that has to be daily consumed to have observed adverse effects is very high for a standard uncertainty factor of 100; hence, we believe that consumption of pheasant tissue coming from fenbendazole-treated pheasants is safe, even when using this drug in an extra-label manner. This finding is reinforced just looking at the interquartile range of the log consumption that is needed to have observable effects (Figure 2), demonstrating that when the risk for presenting effects is low (i.e., the teratogenicity NOAEL, which is the highest one in this study), all three limit distributions could be used, as all of them could be considered safe. Thus, tolerance could be used as a best-practice limit, and this would yield a shorter withdrawal interval because tolerance is the highest limit. On the other hand, when having a higher risk for presenting effects, that is, when applying the carcinogenicity NOAEL (the lowest NOAEL in this study), the limit that should be used to set a withdrawal interval is the LOD, as it is the most conservative parameter. In this case, the withdrawal interval would be longer, as the LOD is the lowest limit. All these points should be taken into account when fenbendazole in pheasants is used extra-label, as, depending on the risk, a withdrawal period should be set up according to the established cutoff. Moreover, when performing the stochastic process and analyzing the correlation coefficients obtained in the sensitivity analysis (Figure 3), findings show that the LOD has a very high negative correlation with the observed adverse effects; therefore, it is the factor that has the strongest influence on having adverse effects after consuming pheasant tissue with fenbendazole sulfone residues. This implies that a small variation in the LOD may have a large impact on the grams of pheasant tissue with fenbendazole sulfone residues that can be safely consumed. Consequently, the limit that defines the use of this drug in an extra-label way has a great impact on the appearance of effects due to the consumption of pheasant tissue with residues. We found similar results when using the deterministic approach to analyze if consuming pheasant tissue with fenbendazole sulfone residues was safe. In the most conservative scenario, which is the different pheasant LODs, the conclusion is that consuming pheasant tissue is safe even when using A Ufs = 1,000 (Supplementary Tables 1, 2). Also, some values for the different tolerances and MRLs are considered not safe, as their values are ≥1. However, these are extreme worst-case scenarios very unlikely to occur. Thus, it can be concluded that consuming pheasant tissue from animals that were previously treated with fenbendazole is safe from a public health perspective.

However, according to the Center for Veterinary Medicine residue tolerances for post-marketing monitoring, the development of safe concentrations values of fenbendazole sulfone in food and withdrawal periods should be established (33). In this study, a broad range of pheasant tissue consumption and limits were analyzed by applying different types of distributions to carry out a stochastic process (Table 5). In the most likely scenario, with Ufs of 100, it can be observed that applying all the consumption ranges and using different limits, pheasant tissue consumption is safe, as almost 95% confidence interval and the means are below 100. However, human food safety values for the consumption of pheasant tissue with fenbendazole sulfone residues for carcinogenicity adverse effects were higher than 100, applying the distribution of the tolerances and the US pheasant consumption. Nevertheless, taking into account that it is probable that the consumption of pheasant tissue in the USA and in the EU is lower than the one used in this study and that the EMA study stated that adverse effects for carcinogenicity were not observed, this scenario, even when applying a Ufs of 100, is also very unlikely to happen.

Our main study limitations were associated with the quality and availability of the data to generate the input values of the model. For example, there were no data on pheasant consumption in the USA, so we extrapolated pheasant consumption based on turkey consumption in the USA. Pheasant consumption in the USA is likely much lower than turkey meat consumption; therefore, our model is likely overestimating the risk for pheasant or representing only the worst-case scenario. Moreover, fenbendazole sulfone residue limits in pheasant meat for human consumption are not established in the USA or in other countries except the EU, which establishes the same limit for all food-producing species except fish. To overcome these two limitations, a stochastic analysis was conducted, taking into account, on the one hand, different consumption of pheasant and turkey meat and, on the other hand, different limits established for poultry, as a similar fenbendazole metabolism is assumed. With this stochastic analysis, we have taken into account the variability and uncertainty that our model has, and those input distributions will likely capture the range of values applicable to pheasants. However, it will be valuable for future studies to have real pheasant consumption data in the USA to more accurately estimate the potential exposure in the USA population and to continue studying other aspects related to drug residues coming from these species.

In conclusion, our results suggest that human consumption of products from ring-necked pheasants treated with fenbendazole in an extra-label manner is safe. With this study, after knowing the possible risks that the consumption of pheasant tissue containing fenbendazole sulfone residues can entail, the use of fenbendazole in pheasants can be established in the future. Also, there is a continuing need for monitoring and surveillance to ensure that Food Safety and Inspection Service program goals are met. The extra-label drug use of fenbendazole in pheasants is very important, as it has shown high efficacy against all helminthiasis stages and against a wide range of helminthiasis.

## Data Availability Statement

The original contributions generated for this study are included in the article/Supplementary Material, further inquiries can be directed to the corresponding author/s.

## Author Contributions

LAT and BM-L contributed to conception and design of the study. MC performed the statistical analysis and wrote the manuscript. All authors contributed to manuscript revision, read, and approved the submitted version.

## Conflict of Interest

The authors declare that the research was conducted in the absence of any commercial or financial relationships that could be construed as a potential conflict of interest.

## Abbreviations

ADI: Acceptable Daily Intake
bw: Bodyweight
EMA: European Medicines Agency
FDA: US Food and Drug Administration
KSU: Kansas State University
LOD: Limit of Detection
MRL: Maximum Residue Limit
NHANES: National Health and Nutrition Examination Survey
NOAEL: No-observed-adverse-effect levels
OIE: World Organization for Animal Health
ppb: parts-per-billion
ppm: parts-per-million
Ufs: Standard Uncertainty Factor.
