# Supplementary material for: Risk Assessment of Human Consumption of Meat From Fenbendazole-Treated Pheasants
Source: Front Vet Sci. 2021 Jun 4;8:665357. doi: 10.3389/fvets.2021.665357 (PMC8212976; doi:10.3389/fvets.2021.665357)
Supplement: Supplementary file 1 [file Data_Sheet_1.docx]

**SUPLEMENTARY MATERIAL**


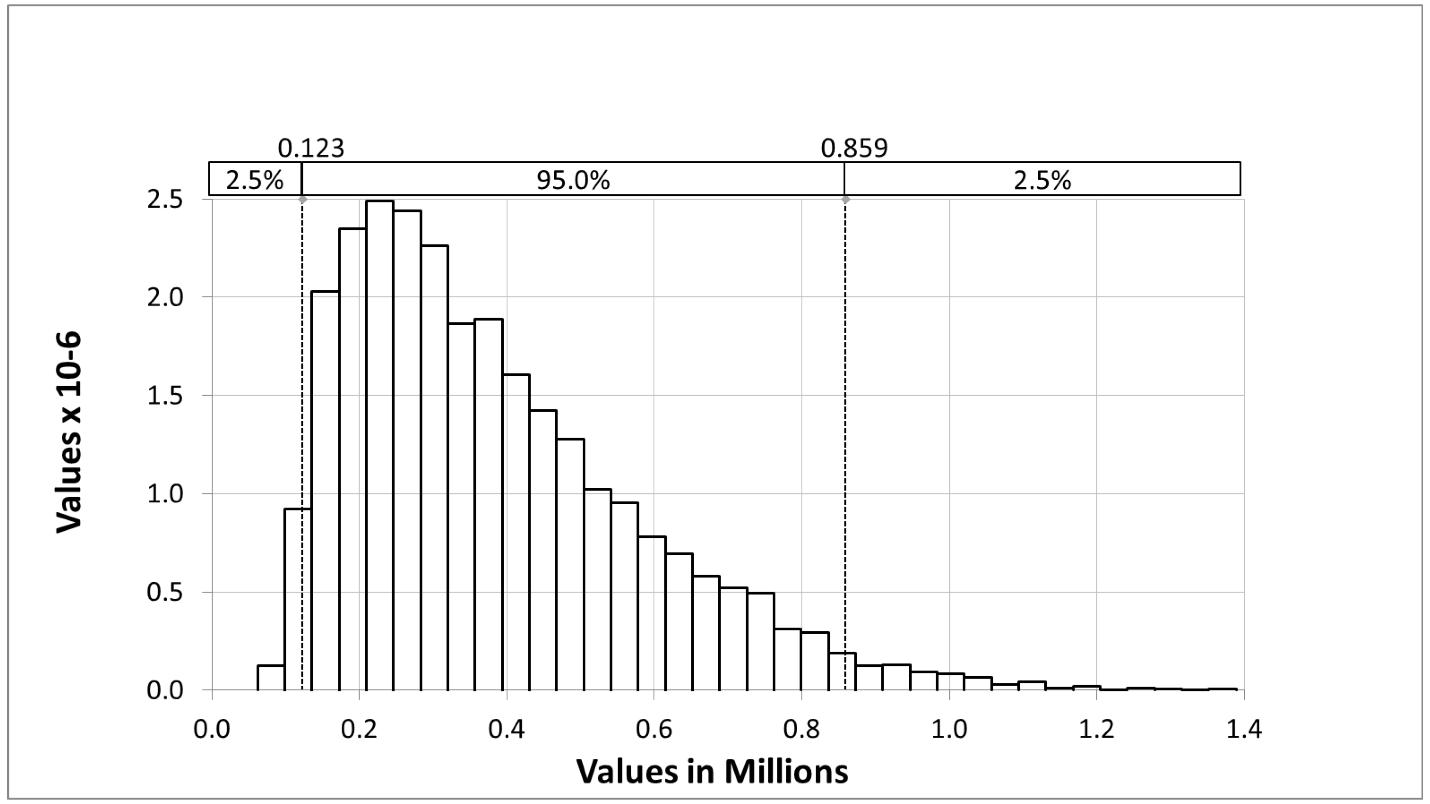


**Supplementary Figure 1**. Pheasant tissue with fenbendazole sulfone residues consumption to have observed effects in grams/day, applying a stochastic process of a pheasant LOD distribution using repeat-dose NOAEL (4 mg/kg bw/day).

**Supplementary Table 1.** Calculation of safe values for human food safety of eating poultry tissue from animals that were previously treated with fenbendazole applying different limits and USA turkey muscle consumption and FDA food consumption values for edible tissues. Values in bold are considered unsafe (≥ 1). RD = repeat-dose, R = reproductive, T = teratogenicity, C = carcinogenicity, Ufs = Standard Uncertainty Factor, MRL = maximum residue limit, LOD = limit of detection.

|  |  |  | **Pop. in USA (turkeys)** | | | | **FDA muscle consumption** | | | | **FDA liver consumption** | | | |
| --- | --- | --- | --- | --- | --- | --- | --- | --- | --- | --- | --- | --- | --- | --- |
|  |  | **Effect** | **RD** | **R** | **T** | **C** | **RD** | **R** | **T** | **C** | **RD** | **R** | **T** | **C** |
| **Tissue-Ufs** | **Species** | **Country-reference limit** |  |  |  |  |  |  |  |  |  |  |  |  |
| **Liver** | Chicken | USA-Tolerance |  |  |  |  |  |  |  |  |  |  |  |  |
| Ufs = 10 |  |  | 0.03 | 0.01 | <0.01 | 0.15 | 0.07 | 0.02 | 0.01 | 0.37 | 0.02 | 0.01 | <0.01 | 0.12 |
| Ufs = 100 |  |  | 0.26 | 0.07 | 0.04 | ***1.48*** | 0.65 | 0.17 | 0.10 | ***3.71*** | 0.22 | 0.06 | 0.03 | ***1.24*** |
| Ufs = 1000 |  |  | ***2.59*** | 0.69 | 0.41 | ***14.80*** | ***6.50*** | ***1.73*** | ***1.04*** | ***37.14*** | ***2.17*** | 0.58 | 0.35 | ***12.38*** |
| **Liver** | Turkey | USA-Tolerance |  |  |  |  |  |  |  |  |  |  |  |  |
| Ufs = 10 |  |  | 0.03 | 0.01 | <0.01 | 0.17 | 0.08 | 0.02 | 0.01 | 0.43 | 0.03 | 0.01 | 0.00 | 0.14 |
| Ufs = 100 |  |  | 0.30 | 0.08 | 0.05 | ***1.71*** | 0.75 | 0.20 | 0.12 | ***4.29*** | 0.25 | 0.07 | 0.04 | ***1.43*** |
| Ufs = 1000 |  |  | ***2.99*** | 0.80 | 0.48 | ***17.08*** | ***7.50*** | ***2.00*** | ***1.20*** | ***42.86*** | ***2.50*** | 0.67 | 0.40 | ***14.29*** |
| **Liver** | Duck | Japan-Tolerance |  |  |  |  |  |  |  |  |  |  |  |  |
| Ufs = 10 |  |  | 0.01 | <0.01 | <0.01 | 0.06 | 0.03 | 0.01 | <0.01 | 0.14 | 0.01 | <0.01 | <0.01 | 0.05 |
| Ufs = 100 |  |  | 0.10 | 0.03 | 0.02 | 0.57 | 0.25 | 0.07 | 0.04 | ***1.43*** | 0.08 | 0.02 | 0.01 | 0.48 |
| Ufs = 1000 |  |  | ***1.00*** | 0.27 | 0.16 | ***5.69*** | ***2.50*** | 0.67 | 0.40 | ***14.29*** | 0.83 | 0.22 | 0.13 | ***4.76*** |
| **Liver** | Duck | New Zealand-Tolerance |  |  |  |  |  |  |  |  |  |  |  |  |
| Ufs = 10 |  |  | <0.01 | <0.01 | <0.01 | <0.01 | <0.01 | <0.01 | <0.01 | 0.01 | <0.01 | <0.01 | <0.01 | <0.01 |
| Ufs = 100 |  |  | <0.01 | <0.01 | <0.01 | 0.03 | 0.01 | <0.01 | <0.01 | 0.07 | <0.01 | <0.01 | <0.01 | 0.02 |
| Ufs = 1000 |  |  | 0.05 | 0.01 | 0.01 | 0.28 | 0.13 | 0.03 | 0.02 | 0.71 | 0.04 | 0.01 | 0.01 | 0.24 |
| **Liver** | Duck | Turkey-Tolerance |  |  |  |  |  |  |  |  |  |  |  |  |
| Ufs = 10 |  |  | <0.01 | <0.01 | <0.01 | <0.01 | <0.01 | <0.01 | <0.01 | <0.01 | <0.01 | <0.01 | <0.01 | <0.01 |
| Ufs = 100 |  |  | <0.01 | <0.01 | <0.01 | 0.01 | 0.01 | <0.01 | <0.01 | 0.04 | <0.01 | <0.01 | <0.01 | 0.01 |
| Ufs = 1000 |  |  | 0.02 | 0.01 | <0.01 | 0.14 | 0.06 | 0.02 | 0.01 | 0.36 | 0.02 | 0.01 | <0.01 | 0.12 |
| **Liver** | All | EU-MRL |  |  |  |  |  |  |  |  |  |  |  |  |
| Ufs = 10 |  |  | <0.01 | <0.01 | <0.01 | 0.01 | 0.01 | <0.01 | <0.01 | 0.04 | <0.01 | <0.01 | <0.01 | 0.01 |
| Ufs = 100 |  |  | 0.02 | 0.01 | <0.01 | 0.14 | 0.06 | 0.02 | 0.01 | 0.36 | 0.02 | 0.01 | <0.01 | 0.12 |
| Ufs = 1000 |  |  | 0.25 | 0.07 | 0.04 | ***1.42*** | 0.63 | 0.17 | 0.10 | ***3.57*** | 0.21 | 0.06 | 0.03 | ***1.19*** |
| **Liver** | Pheasant | USA-LOD |  |  |  |  |  |  |  |  |  |  |  |  |
| Ufs = 10 |  |  | <0.01 | <0.01 | <0.01 | <0.01 | <0.01 | <0.01 | <0.01 | <0.01 | <0.01 | <0.01 | <0.01 | <0.01 |
| Ufs = 100 |  |  | <0.01 | <0.01 | <0.01 | 0.01 | 0.01 | <0.01 | <0.01 | 0.03 | <0.01 | <0.01 | <0.01 | 0.01 |
| Ufs = 1000 |  |  | 0.02 | 0.01 | <0.01 | 0.11 | 0.05 | 0.01 | 0.01 | 0.29 | 0.02 | <0.01 | <0.01 | 0.10 |
| **Pectoral muscle** | Pheasant | USA-LOD |  |  |  |  |  |  |  |  |  |  |  |  |
| Ufs = 10 |  |  | <0.01 | <0.01 | <0.01 | <0.01 | <0.01 | <0.01 | <0.01 | <0.01 | <0.01 | <0.01 | <0.01 | <0.01 |
| Ufs = 100 |  |  | <0.01 | <0.01 | <0.01 | <0.01 | <0.01 | <0.01 | <0.01 | <0.01 | <0.01 | <0.01 | <0.01 | <0.01 |
| Ufs = 1000 |  |  | <0.01 | <0.01 | <0.01 | 0.01 | 0.01 | <0.01 | <0.01 | 0.03 | <0.01 | <0.01 | <0.01 | 0.01 |
| **Thigh muscle** | Pheasant | USA-LOD |  |  |  |  |  |  |  |  |  |  |  |  |
| Ufs = 10 |  |  | <0.01 | <0.01 | <0.01 | <0.01 | <0.01 | <0.01 | <0.01 | <0.01 | <0.01 | <0.01 | <0.01 | <0.01 |
| Ufs = 100 |  |  | <0.01 | <0.01 | <0.01 | <0.01 | <0.01 | <0.01 | <0.01 | <0.01 | <0.01 | <0.01 | <0.01 | <0.01 |
| Ufs = 1000 |  |  | <0.01 | <0.01 | <0.01 | 0.01 | 0.01 | <0.01 | <0.01 | 0.04 | <0.01 | <0.01 | <0.01 | 0.01 |

**Supplementary Table 2.** Calculation of safe values for human food safety of eating poultry tissue from animals that were previously treated with fenbendazole applying different limits and Belgium pheasant muscle consumption, mean of Belgium pheasant muscle consumption and 95 percentile of pheasant muscle consumption in Belgium. Values in bold are considered unsafe (≥ 1). RD = repeat-dose, R = reproductive, T = teratogenicity, C = carcinogenicity, Ufs = Standard Uncertainty Factor, MRL = maximum residue limit, LOD = limit of detection.

|  |  |  | **Pop. in Belgium (pheasants)** | | | | **Consumers in Belgium (mean)** | | | | **Consumers in Belgium (Perc 95)** | | | |
| --- | --- | --- | --- | --- | --- | --- | --- | --- | --- | --- | --- | --- | --- | --- |
|  |  | **Effect** | **RD** | **R** | **T** | **C** | **RD** | **R** | **T** | **C** | **RD** | **R** | **T** | **C** |
| **Tissue-Ufs** | **Species** | **Country-reference limit** |  |  |  |  |  |  |  |  |  |  |  |  |
| **Liver** | Chicken | USA-Tolerance |  |  |  |  |  |  |  |  |  |  |  |  |
| Ufs = 10 |  |  | <0.01 | <0.01 | <0.01 | <0.01 | 0.02 | <0.01 | <0.01 | 0.09 | 0.03 | 0.01 | <0.01 | 0.15 |
| Ufs = 100 |  |  | <0.01 | <0.01 | <0.01 | <0.01 | 0.17 | 0.04 | 0.03 | 0.95 | 0.26 | 0.07 | 0.04 | ***1.47*** |
| Ufs = 1000 |  |  | 0.01 | <0.01 | <0.01 | 0.04 | ***1.65*** | 0.44 | 0.26 | ***9.46*** | ***2.58*** | 0.69 | 0.41 | ***14.73*** |
| **Liver** | Turkey | USA-Tolerance |  |  |  |  |  |  |  |  |  |  |  |  |
| Ufs = 10 |  |  | <0.01 | <0.01 | <0.01 | <0.01 | 0.02 | 0.01 | <0.01 | 0.11 | 0.03 | 0.01 | <0.01 | 0.17 |
| Ufs = 100 |  |  | <0.01 | <0.01 | <0.01 | <0.01 | 0.19 | 0.05 | 0.03 | ***1.09*** | 0.30 | 0.08 | 0.05 | ***1.70*** |
| Ufs = 1000 |  |  | 0.01 | <0.01 | <0.01 | 0.05 | ***1.91*** | 0.51 | 0.31 | ***10.91*** | ***2.98*** | 0.79 | 0.48 | ***17.00*** |
| **Liver** | Duck | Japan-Tolerance |  |  |  |  |  |  |  |  |  |  |  |  |
| Ufs = 10 |  |  | <0.01 | <0.01 | <0.01 | <0.01 | 0.01 | <0.01 | <0.01 | 0.04 | 0.01 | <0.01 | <0.01 | 0.06 |
| Ufs = 100 |  |  | <0.01 | <0.01 | <0.01 | <0.01 | 0.06 | 0.02 | 0.01 | 0.36 | 0.10 | 0.03 | 0.02 | 0.57 |
| Ufs = 1000 |  |  | <0.01 | <0.01 | <0.01 | 0.02 | 0.64 | 0.17 | 0.10 | ***3.64*** | 0.99 | 0.26 | 0.16 | ***5.67*** |
| **Liver** | Duck | New Zealand-Tolerance |  |  |  |  |  |  |  |  |  |  |  |  |
| Ufs = 10 |  |  | <0.01 | <0.01 | <0.01 | <0.01 | <0.01 | <0.01 | <0.01 | <0.01 | <0.01 | <0.01 | <0.01 | <0.01 |
| Ufs = 100 |  |  | <0.01 | <0.01 | <0.01 | <0.01 | <0.01 | <0.01 | <0.01 | 0.02 | <0.01 | <0.01 | <0.01 | 0.03 |
| Ufs = 1000 |  |  | <0.01 | <0.01 | <0.01 | <0.01 | 0.03 | 0.01 | 0.01 | 0.18 | 0.05 | 0.01 | 0.01 | 0.28 |
| **Liver** | Duck | Turkey-Tolerance |  |  |  |  |  |  |  |  |  |  |  |  |
| Ufs = 10 |  |  | <0.01 | <0.01 | <0.01 | <0.01 | <0.01 | <0.01 | <0.01 | <0.01 | <0.01 | <0.01 | <0.01 | <0.01 |
| Ufs = 100 |  |  | <0.01 | <0.01 | <0.01 | <0.01 | <0.01 | <0.01 | <0.01 | 0.01 | <0.01 | <0.01 | <0.01 | 0.01 |
| Ufs = 1000 |  |  | <0.01 | <0.01 | <0.01 | <0.01 | 0.02 | <0.01 | <0.01 | 0.09 | 0.02 | 0.01 | <0.01 | 0.14 |
| **Liver** | All | EU-MRL |  |  |  |  |  |  |  |  |  |  |  |  |
| Ufs = 10 |  |  | <0.01 | <0.01 | <0.01 | <0.01 | <0.01 | <0.01 | <0.01 | 0.01 | <0.01 | <0.01 | <0.01 | 0.01 |
| Ufs = 100 |  |  | <0.01 | <0.01 | <0.01 | <0.01 | 0.02 | <0.01 | <0.01 | 0.09 | 0.02 | 0.01 | <0.01 | 0.14 |
| Ufs = 1000 |  |  | <0.01 | <0.01 | <0.01 | <0.01 | 0.16 | 0.04 | 0.03 | 0.91 | 0.25 | 0.07 | 0.04 | ***1.42*** |
| **Liver** | Pheasant | USA-LOD |  |  |  |  |  |  |  |  |  |  |  |  |
| Ufs = 10 |  |  | <0.01 | <0.01 | <0.01 | <0.01 | <0.01 | <0.01 | <0.01 | <0.01 | <0.01 | <0.01 | <0.01 | <0.01 |
| Ufs = 100 |  |  | <0.01 | <0.01 | <0.01 | <0.01 | <0.01 | <0.01 | <0.01 | 0.01 | <0.01 | <0.01 | <0.01 | 0.01 |
| Ufs = 1000 |  |  | <0.01 | <0.01 | <0.01 | <0.01 | 0.01 | <0.01 | <0.01 | 0.07 | 0.02 | 0.01 | <0.01 | 0.11 |
| **Pectoral muscle** | Pheasant | USA-LOD |  |  |  |  |  |  |  |  |  |  |  |  |
| Ufs = 10 |  |  | <0.01 | <0.01 | <0.01 | <0.01 | <0.01 | <0.01 | <0.01 | <0.01 | <0.01 | <0.01 | <0.01 | <0.01 |
| Ufs = 100 |  |  | <0.01 | <0.01 | <0.01 | <0.01 | <0.01 | <0.01 | <0.01 | <0.01 | <0.01 | <0.01 | <0.01 | <0.01 |
| Ufs = 1000 |  |  | <0.01 | <0.01 | <0.01 | <0.01 | <0.01 | <0.01 | <0.01 | 0.01 | <0.01 | <0.01 | <0.01 | 0.01 |
| **Thigh muscle** | Pheasant | USA-LOD |  |  |  |  |  |  |  |  |  |  |  |  |
| Ufs = 10 |  |  | <0.01 | <0.01 | <0.01 | <0.01 | <0.01 | <0.01 | <0.01 | <0.01 | <0.01 | <0.01 | <0.01 | <0.01 |
| Ufs = 100 |  |  | <0.01 | <0.01 | <0.01 | <0.01 | <0.01 | <0.01 | <0.01 | <0.01 | <0.01 | <0.01 | <0.01 | <0.01 |
| Ufs = 1000 |  |  | <0.01 | <0.01 | <0.01 | <0.01 | <0.01 | <0.01 | <0.01 | 0.01 | <0.01 | <0.01 | <0.01 | 0.01 |

**Supplementary Table 3.** Comparison of three stochastic processes to calculate the mean and 95% CI of consumption (grams/day) of pheasant tissues contaminated with fenbendazole sulfone residues to have observed adverse effects. MRL = maximum residue limit, LOD = limit of detection, NOAEL = No-observed-adverse-effect level.

| *Tissue consumption to have observed adverse effects (mean (g/day) [95% CI])* | | | |
| --- | --- | --- | --- |
|  | **Reference limits distribution** | | |
| **NOAEL** | **Tolerances** | **MRLs** | **LODs** |
| **Repeat-dose**  **(4 ppm)** | 2,505  [418, 15,710] | 6,238  [3,850, 9,640] | 382,965  [122,956, 859,488] |
| **Reproductive**  **(15 ppm)** | 9,401  [1,587, 59,318] | 23,386  [14,451, 35,977] | 1,370,884  [432,340, 3,125,947] |
| **Teratogenicity (25 ppm)** | 15,687  [2,615, 93,525] | 38,949  [24,021, 59,849] | 2,281,479  [714,942, 5,138,513] |
| **Carcinogenicity (0.7 ppm)** | 442  [73, 2,785] | 1,090  [690, 1,650] | 63,932  [19,859, 146,263] |
